# Flexural Strength Evaluation of Multi-Cell Composite T-Shaped Concrete-Filled Steel Tubular Beams

**DOI:** 10.3390/ma14112838

**Published:** 2021-05-26

**Authors:** Yanfei Shen, Yongqing Tu

**Affiliations:** 1 School of Civil and Architectural Engineering, Wuyi University, Jiangmen 529020, China; shenyanfei@wyu.edu.cn; 2 Department of Civil Engineering, Beihang University, Beijing 100191, China

**Keywords:** steel tube, concrete-filled, T-shaped, composite, flexural strength, unified theory, plastic stress distribution

## Abstract

The multi-cell composite T-shaped concrete-filled steel tubular (MT-CFST) element is an innovative structural form. It has great potential for construction applications because of favorable advantages over traditional composite elements. The flexural strength of MT-CFST beams was investigated in this study to provide recommendations in line with existing design codes. First, formulations to evaluate the flexural strength of MT-CFST beams were derived based on the Unified Theory and plastic stress distribution method (PSDM). For the Unified Theory-based formula, a modified confinement effect factor that considers the shape of a cross-section was proposed. An experimental study on the flexural behavior of six MT-CFST beams as well as two hollow section counterparts was conducted. The influence of bending moment direction, concrete infill, wall thickness, and cross-section sizes were investigated. The accuracy of the proposed formulations was verified against the test results and numerical results from finite element modeling. The comparisons showed that the formula in line with the Unified Theory provided more accurate predictions with reasonable conservatism for the studied MT-CFST beams.

## 1. Introduction

**Figure 1:**
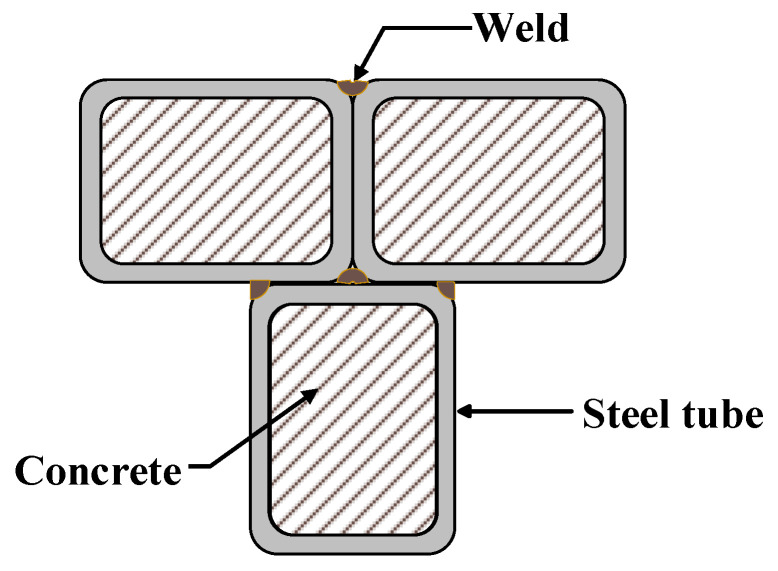
A typical MT-CFST cross-section.

Concrete-filled steel tubular (CFST) elements can reduce the materials costs and the construction time effectively in comparison to equivalent steel or reinforced concrete elements, since CFST cross-sections make full use of the advantages of both steel and concrete materials [1,2,3,4,5]. Recently, the applications of special-shaped (L, T, †, etc.) CFST cross-sections in residential buildings and bridges have attracted much attention [6,7,8,9,10,11,12]. With favorable static and seismic performance, multi-cell composite T-shaped concrete-filled steel tubular (MT-CFST) elements, first proposed by Tu et al. [7,8,9], have great potential for construction applications. A typical MT-CFST cross-section is shown in Figure 1, in which three rectangular (square) steel tubes are first joined by fillet weld, and concrete is then poured into the steel tubes.

Design provisions for normal CFST members have been established in a series of design codes such as AISC 360-16 [13], EC4 [14] and GB 50936-2014 [15]. However, no design guidelines are provided for the implementation of special-shaped CFST members, even though GB50936-2014 [15] provides design guidelines for octagon and hexadecagon CFST sections. Currently, the limited knowledge regarding the actual mechanical behavior of special-shaped CFST members is a major deterrent to the establishment of related design provisions and their widespread use.

For the determination of flexural strength of CFST beams with compact sections, the plastic stress distribution method (PSDM) is adopted in both AISC 360-16 [13] and EC4 [14]. This method assumes a CFST section undergoes full plasticization under an ultimate limit state, in which steel reaches yield strength (*f_y_*) in both compression and tension regions, and the concrete compressive stress is equal to a shape factor times the compressive strength (*f_c_**^’^***) of concrete. The tensile strength of concrete is neglected in establishing the internal force equilibrium of the section. Differently from AISC 360-16 [13] and EC4 [14], GB 50936-2014 [15] adopts a Unified Theory to predict the ultimate capacity of CFST members under different loadings. The Unified Theory was first proposed by Zhong [16], and it was continuously improved due to the great effort of Han [17]. In the Unified Theory, steel is integrated with concrete infill, and they are taken as a new composite material. A unified body is then used to calculate the ultimate capacities of CFST members under different loading conditions.

Previous studies on the evaluation of flexural strength of CFST beams were mainly focused on normal sections. The studies of Jiang et al. [18], Elchalakani et al. [19], GHo and Liu [20], Xiong et al. [21], and Chitawadagi and Narasimhan [22] showed PSDM was found to underpredict the flexural strength in almost all cases in comparison with experimental results of CFST beams with circular, square and rectangular cross-sections. In addition, Ren et al. [23] reported that PSDM was conservative for CFST beams with elliptical cross-sections. A series of studies showed that the predicted results from the simplified formulations in line with the Unified Theory were in close agreement with test results of circular CFST beams, but the Unified Theory-based formulations produced unconservative errors for square and rectangular CFST beams with low concrete-to-steel rations in a few cases [24,25,26,27,28]. With respect to composite CFST beams, Moon et al. [10] reported PSDM gave a reasonably conservative prediction for the bending resistance of a T-shaped composite CFST girder composed of CFST circular beams and concrete slabs under both positive and negative bending moments, based on experimental and numerical studies. Since the MT-CFST section is not covered in the existing design standards [13,14,15], the accuracy of design provisions in line with the Unified Theory of concrete-filled steel tubes, and the plastic stress distribution method (PSDM) for MT-CFST members should be assessed. 

The following sections of the paper are mainly focused on three aspects: (1) deriving a Unified Theory-based formula applicable to MT-CFST beams by means of introducing a modified confinement effect factor, (2) conducting an experimental study on the flexural behavior of six MT-CFST beams as well as two hollow section counterparts, and (3) evaluating the accuracy of the Unified Theory-based method and plastic stress distribution method (PSDM) for the determination of the flexural strength of MT-CFST beams.

## 2. Methods to Predict the Flexural Strength of MT-CFST Beams

### 2.1. The Nominal Bending Resistance Determined by the Unified Theory

In accordance with the Unified Theory, the nominal ultimate bending resistance of a CFST beam is determined by

(1) $$M_{u}=\gamma_{m}W_{sc}f_{sc}$$

where *W_sc_* is the elastic section modulus; *f_sc_* is the compressive strength of the composite section; *γ_m_* is the flexural strength factor for the composite material that exhibits non-isotropic strength behavior (the compressive strength of this composite material is higher than its tensile strength); γ_m_, provided in [9] for T-shaped CFST sections, given by Equations (2) and (3), is adopted in this study.

(2) $$\gamma_{m}=0.51+0.47\ln\left(\alpha f_{y}/f_{c}^{\prime }+0.52\right)\;\mathrm{for}\;\mathrm{positive}\;\mathrm{bending}$$

(3) $$\gamma_{m}=0.84+0.78\ln\left(\alpha f_{y}/f_{c}^{\prime }+0.53\right)\;\mathrm{for}\;\mathrm{negative}\;\mathrm{bending}$$

where *f_y_* and *f_c_^’^* are the yield strength of the steel tube and the compressive strength (prism) of the concrete infill, respectively; *α* = (A_s_^f^/A_c_^f^ + A_s_^w^/A_c_^w^); A_s_^f^ and A_s_^w^ are steel area of the flange and the web, respectively; A_c_^f^ and A_c_^w^ are concrete area of the flange and the web, respectively.

A unified formula to calculate *f_sc_* for CFST polygon sections is provided in GB 50936-2014 [15]. It was developed by regression analysis and calibration against experimental results, given by

(4) $$f_{sc}=\left(1.212+B\xi +C\xi {\;}^{2}\right)f_{c}^{\prime }$$

(5) $$\xi =\frac{A_{s}f_{y}}{A_{c}f_{c}^{\prime }}$$

where *A_s_* and *A_c_* are the area of the steel and concrete infill of the composite section, respectively. The coefficient B and C consider the contributions of steel and concrete, respectively. The confinement factor *ξ*, derived from the CFST circular and square sections, is used to account for the confinement effect on concrete infill. The factor *ξ* works well for CFST circular and square sections [29,30], but it cannot appropriately reflect the confinement effect for a MT-CFST section [9], since the confinement effect has a great relationship with the shape of the cross-section. Thus, a modified confinement factor (*ξ_MT_*) and a new approach of calculating *f_sc_* for MT-CFST sections were developed in this study. The development of *ξ_MT_* and the calculation of *f_sc_* for an MT-CFST section are described in the following subsubsections.

#### 2.1.1. A Modified Confinement Factor Considering Geometry Effects on Confinement

**Figure 2:**
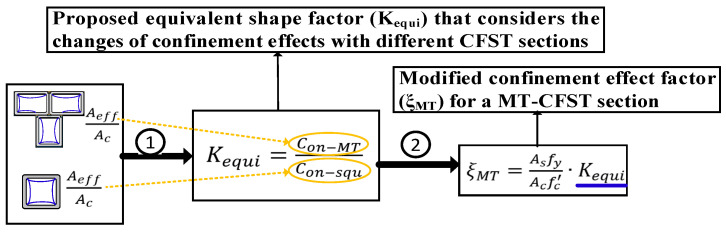
Illustration of the development of the modified confinement factor (*ξ_MT_*).

**Figure 3:**
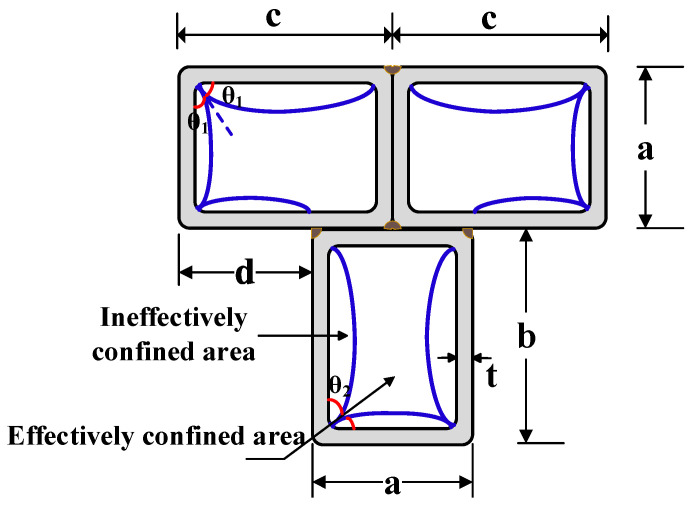
Division of effectively and ineffectively confined area for infilled concrete.

The development of the modified confinement factor (*ξ_MT_*) is illustrated in Figure 2, described as follows:

First, based on the research findings of Zhang et al. [29] and Mander et al. [31], the in-filled concrete of a multi-cell T-shaped composite section is divided into effectively and ineffectively confined areas through a second-degree parabola with an initial tangent angle of *θ*, as shown in Figure 3. The purpose of dividing effectively and ineffectively confined areas for in-filled concrete is to obtain the modified confinement factor (*ξ_MT_*) that considers the effect of the cross-section shape on the confinement to in-filled concrete. The division of effectively and ineffectively confined concrete areas depends on the shape of the steel tube in the outermost layer. Confining stress from the steel tube in the effectively confined core is larger than that in the ineffectively confined area.

Second, a confinement ratio (*C_on_*) is defined, as given by Equation (6):

(6) $$C_{on}=\frac{A_{eff}}{A_{c}}$$

where *A_eff_* and *A_c_* are the area of the effectively confined core and the total area of the concrete infill, respectively. To simplify the calculation process, the round corner of all of the hollow sections is taken as sharp. The *C_on_* for different components of an MT-CFST section can be obtained by integration, given by

(7) $$C_{on-MT-f}\approx 1-\frac{s}{6}\left(1+\frac{1}{s}+\frac{1}{m}\right)\tan\theta_{1}\;\mathrm{for}\;\mathrm{the}\;\mathrm{flange}\;\mathrm{of}\;\mathrm{an}\;\mathrm{MT}-\mathrm{CFST}\;\mathrm{section}$$

(8) $$C_{on-MT-w}\approx 1-\frac{1+2n^{2}}{6n}\tan\theta_{2}\;\mathrm{for}\;\mathrm{the}\;\mathrm{web}\;\mathrm{of}\;\mathrm{an}\;\mathrm{MT}-\mathrm{CFST}\;\mathrm{section}$$

where *s* = (*c* − 2t)/(*a* − 2*t*); *m* = (*c* − 2*t*)/*d*; *n* = (*b* − 2*t*)/(*a* − 2*t*); *θ*_1_ = min {45°, 180°/π∙arctan(1/s)}; *θ*_2_ = min{45°, 180°/π∙arctan(1/*n*)}.

Third, assuming that under the same confinement ratio the enhancement effect is only influenced by the geometry of a cross-section, an equivalent shape factor (*K_equi_*) that considers the changes of confinement effects with different CFST sections was proposed. Based on the relationship between the confinement ratio of the flange (web) of a T-shaped section and that of a square section, the equivalent shape factor is given by

(9) $$K_{equi-f}=\frac{C_{on-MT-f}}{C_{on-squ}}\;\mathrm{for}\;\mathrm{the}\;\mathrm{flange}\;\mathrm{of}\;\mathrm{an}\;\mathrm{MT}-\mathrm{CFST}\;\sec\mathrm{tion}$$

(10) $$K_{equi-w}=\frac{C_{on-MT-w}}{C_{on-squ}}\;\mathrm{for}\;\mathrm{the}\;\mathrm{web}\;\mathrm{of}\;\mathrm{an}\;\mathrm{MT}-\mathrm{CFST}\;\sec\mathrm{tion}$$

where *C_on-squ_* is the confinement ratio of the corresponding square section (the square section has the same area and wall thickness as the flange (web) of the T-shaped section), given by

(11) $$C_{on-squ}=1-\frac{2}{3}\tan\theta \;$$

where *θ* is equal to *θ*_1_ for the calculation of *K__equi-f_*, and *θ*_2_ for *K__equi-w_*.

Lastly, the modified confinement factor (*ξ_MT_*) for the flange and web of an MT-CFST section is given by

(12) $$\xi_{MT-f}=K_{equi-f}\left(A_{s}^{f}f_{y}/A_{c}^{f}f_{c}^{\prime }\right)\;\mathrm{for}\;\mathrm{the}\;\mathrm{flange}\;\mathrm{of}\;\mathrm{an}\;\mathrm{MT}-\mathrm{CFST}\;\sec\mathrm{tion}$$

(13) $$\xi_{MT-w}=K_{equi-w}\left(A_{s}^{w}f_{y}/A_{c}^{w}f_{c}^{\prime }\right)\;\mathrm{for}\;\mathrm{the}\;\mathrm{web}\;\mathrm{of}\;\mathrm{an}\;\mathrm{MT}-\mathrm{CFST}\;\sec\mathrm{tion}$$

where *A_s_^f^* and *A_s_^w^* are steel area of the flange and the web, respectively, and *A_c_^f^* and *A_c_^w^* are concrete area of the flange and the web, respectively.

#### 2.1.2. Calculation of *f_sc_* for an MT-CFST Section

Assumes that the compressive strength of an MT-CFST section can be determined by adding the compressive strength of its components, as given by

(14) $$Af_{sc}=A_{f}f_{sc-f}+A_{w}f_{sc-w}$$

Rewriting Equation (14) gives

(15) $$f_{sc}=\frac{A_{f}f_{sc-f}+A_{w}f_{sc-w}}{A_{f}+A_{w}}$$

where *A*, *A_f_* and *A_w_* are the area of the whole section, flange and web, respectively; *A* = *A_f_* + *A_w_*, and *f_sc-f_* and *f_sc-w_* are the compressive strength of the flange component and web component, respectively. *f_sc-f_* and *f_sc-w_* are calculated based on the unified formula using a modified confinement factor (*ξ_MT_*) that considers the geometry effects on confinement, given by

(16) $$f_{sc-f}=[1.212+B\xi_{MT-f}+C{\left(\xi_{MT-f}\right)}^{2}]f_{c}^{\prime }$$

(17) $$f_{sc-w}=[1.212+B\xi_{MT-w}+C{\left(\xi_{MT-w}\right)}^{2}]f_{c}^{\prime }$$

where the coefficient *B* and *C* are given by

(18) $$B=0.131\frac{f_{y}}{213}+0.723$$

(19) $$C=-0.7\frac{f_{c}^{\prime }}{14.4}+0.026$$

Substituting Equations (16) and (17) into Equation (7) gives

(20) $$f_{sc}=\left[1.212+\frac{B\left(A_{f}\xi_{MT-f}+A_{w}\xi_{MT-w}\right)}{A_{f}+A_{w}}+\frac{C\left(A_{f}\xi_{MT-f}{}^{2}+A_{w}\xi_{MT-w}{}^{2}\right)}{A_{f}+A_{w}}\right]f_{c}^{\prime }$$

### 2.2. The Nominal Bending Resistance Determined by the Plastic Stress Distribution Method

**Figure 4:**
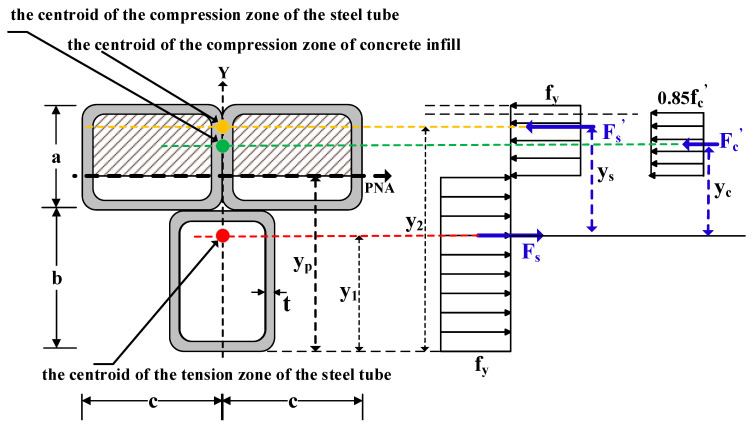
Plastic stress distribution for an MT-CFST compact section under positive bending.

The plastic stress distribution for an MT-CFST compact section under positive bending moment is shown in Figure 4. In this figure, *y_p_* is the distance from the bottom of the cross-section to the plastic neutral axis (PNA); *y_s_* is the distance from the centroid of the tension zone of the steel tube to the centroid of the tension zone of the concrete infill; *y_c_* is the distance from the centroid of the tension zone of the steel tube to the centroid of the steel tube’s compression zone; *y*_1_ is the distance from the bottom of the cross-section to the centroid of the tension zone of the steel tube, and *y*_2_ is the distance from the bottom of the cross-section to the centroid of the compression zone of the steel tube. *F**_s_*** and *F**_s_^’^*** are the tension force and compression force of the steel tube, respectively, and *F**_c_^’^*** is the compression force of the concrete infill. Details on the equations used to obtain *y_p_*, *y_s_*, *y_c_*, *y*_1_ and *y*_2_ are shown in Appendix A. The nominal bending resistance can be obtained through equilibrium equations, given by

(21) $$M_{u}=F_{s}^{\prime }y_{s}+F_{c}^{\prime }y_{c}$$

(22) $$F_{s}^{\prime }=f_{y}A_{s}^{\prime }\;\;$$

(23) $$F_{c}^{\prime }=0.85f_{c}^{\prime }A_{c}^{\prime }$$

where $A_{s}^{\prime }$ and $A_{c}^{\prime }$ are the compression area of steel tube and concrete infill, respectively; Equilibrium equations to obtain $A_{s}^{\prime }$ and $A_{c}^{\prime }$ are shown in Appendix A. For the plastic stress distribution method, the compressive strength of the concrete infill is taken as 0.85$f_{c}^{\prime }$, and the tensile strength of concrete is neglected. Note that EC4 also allows the coefficient 0.85 to be replaced by 1 for normal CFST sections. In the case of an MT-CFST compact section under positive bending moment, the nominal bending resistance can be obtained by using a similar approach.

## 3. Experimental Study

### 3.1. Description of Specimens

The multi-cell composite T-shaped concrete-filled steel tubular (MT-CFST) specimens were designed to be not susceptible to local buckling and lateral-torsional buckling before the composite section reaches full plastic stress distribution. For a CFST beam, the presence of in-filled concrete prevents the hollow steel tube from deforming inward and changes the local buckling mode of the hollow steel tube within the cross-section and along the length of the member [17,24,32]. In this study, the limiting wall slenderness (width-to-thickness ratio) for MT-CFST sections was conservatively taken as that for steel rectangular hollow compact sections. It should be noted that the steel rectangular hollow compact sections here refer to cross-sections that can develop their plastic moment resistance but have limited rotation capacity because of local buckling. The limiting laterally unbraced length for MT-CFST beams was conservatively taken as that of equivalent T-shaped steel beams under the limit of plastic yielding specified in AISC 360-16 [13]. The limiting wall slenderness (for compact, non-compact and slender section) and limiting laterally unbraced length (for plastic yielding and inelastic lateral-torsion buckling) applicable to MT-CFST members will be investigated in the authors’ future paper.

**Figure 5:**
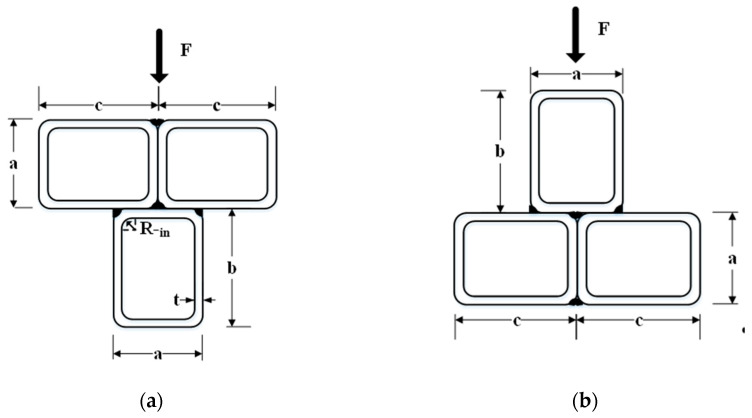
Cross-sections under different bending moments: (**a**) Positive bending moment; (**b**) negative bending moment.

**Figure 6:**
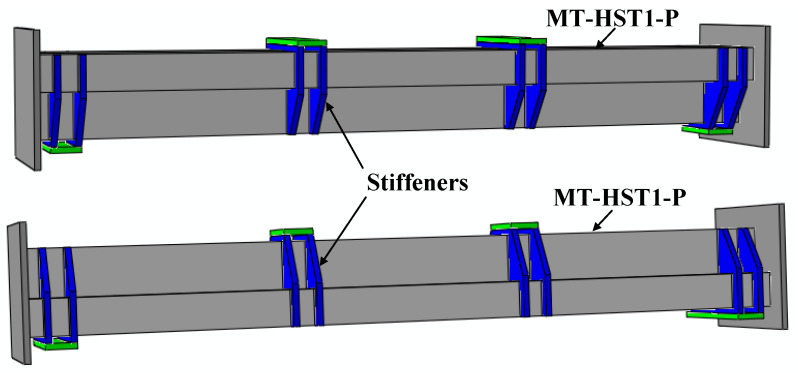
MT-HST specimens with transverse stiffeners.

Six multi-cell composite T-shaped concrete-filled steel tubular (MT-CFST) beams and two multi-cell composite T-shaped hollow steel tubular (MT-HST) beams were tested. A summary of the geometric and material properties of the specimens is shown in Table 1. The specimens were divided into four groups. For each group, two identical specimens were prepared with the same material and geometric properties. To investigate the effect of bending moment direction on the flexural behavior of the specimens, both positive and negative bending moments were considered for each group. The letters P and N in the specimen name correspond to positive bending moment and negative bending moment, respectively. The cross-sectional parameters (*a*, *b*, *c*, *t* and R_-in_) are defined in Figure 5. The letters *a*, *b* and *c* represent the side length of a single steel rectangular hollow section (RHS). The total height of the composite T-shaped cross-section is *a* plus *b*, and 2*c* is the flange width of the composite cross-section. The characters t and R_-in_ are the wall thickness and inside radius of the corner, respectively. The height and wall thickness of the composite cross-section varied for different MT-CFST specimens. The nominal length (L) of all of the specimens was 1300 mm. For steel tubes with the nominal wall thickness of 2.50 mm, the average yield stress (*f_y_*) and average Young’s modulus (E_s_) of steel tube were 315 MPa and 198.2 GPa, respectively, while for those with the nominal wall thickness of 2 mm, *f_y_* and E_s_ were 321.6 MPa and 199.6 GPa, respectively. The average compressive strength (*f_c_^’^*) and average Young’s modulus (E_c_) of the concrete infill for all of the MT-CFST specimens were 41.3 MPa and 35.5 GPa, respectively. For each composite T-shaped hollow steel tubular specimen, four pairs of special transverse stiffeners (shown in Figure 6), which were perpendicular to the flanges and welded to the web, were used to prevent premature local buckling caused by concentrated forces. The fabrication of the specimens was similar to that presented in [8].

The comparison between specimens in the second group (MT-CFST2) and those in the first group (MT-HST1) assessed the contribution of concrete infill to flexural strength. The third group of specimens (MT-CFST3) was compared with the second group of specimens (MT-CFST2) to investigate the effect of wall thickness on flexural strength, whereas the comparison of specimens in the fourth group (MT-CFST4) and those in the second group (MT-CFST2) emphasized the influence of cross-section sizes.

### 3.2. Description of Test Setup

**Figure 7:**
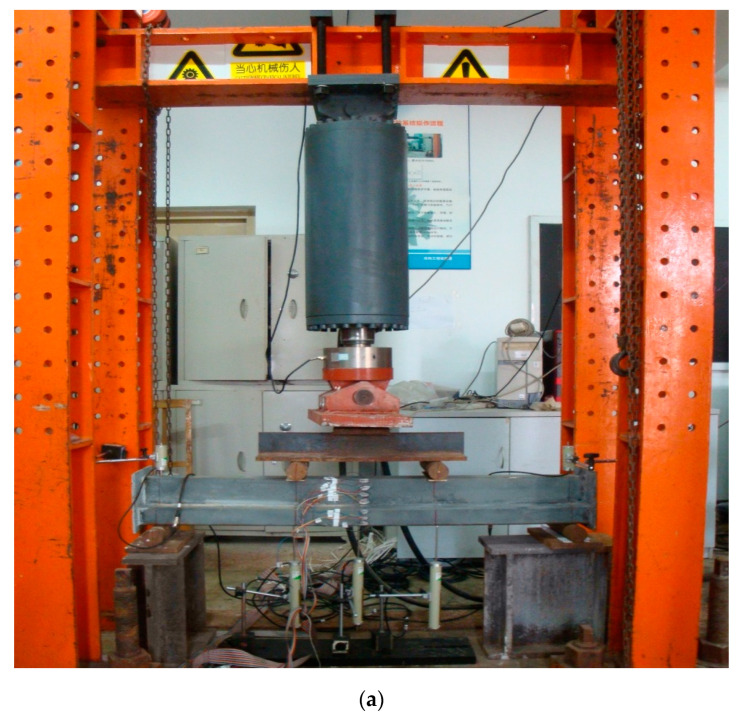

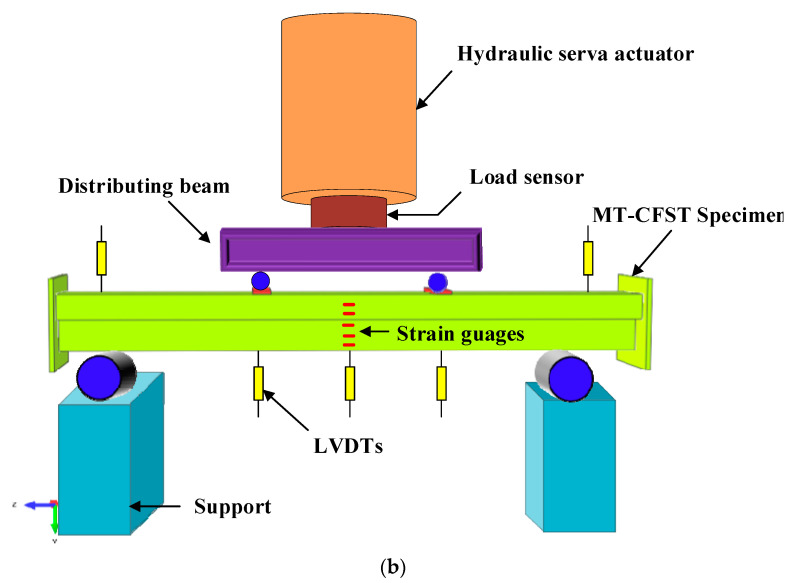
A general view of the four-point bending test and schematic diagram of the test setup: (**a**) Four-point bending test of MT-HST specimen; (**b**) schematic diagram of the test setup.

Four groups of four-point bending tests were conducted at the Structural Engineering Laboratory of Beihang University (BUAA). A hydraulic machine with a capacity of 1000 kN under displacement control was employed. The simply supported span for each specimen was 1200 mm. A general view of the four-point bending test and a schematic diagram of the test setup are shown in Figure 7. For each group, one specimen was subjected to a positive bending moment, while a negative bending moment was applied to the other. All the specimens bent about the unsymmetrical axis. Three linear variable displacement transducers (LVDTs) were used to measure the deflections at the mid-span and at the two loading points. Another two LVDTs were placed at the support points. For MT-CFST specimens, a total of seven strain gauges were attached on the surface of steel tubes in the mid-span, in which two were on the top and bottom of the cross-section, respectively, and five were attached along the height of the cross-section.

### 3.3. Test Results

**Figure 8:**
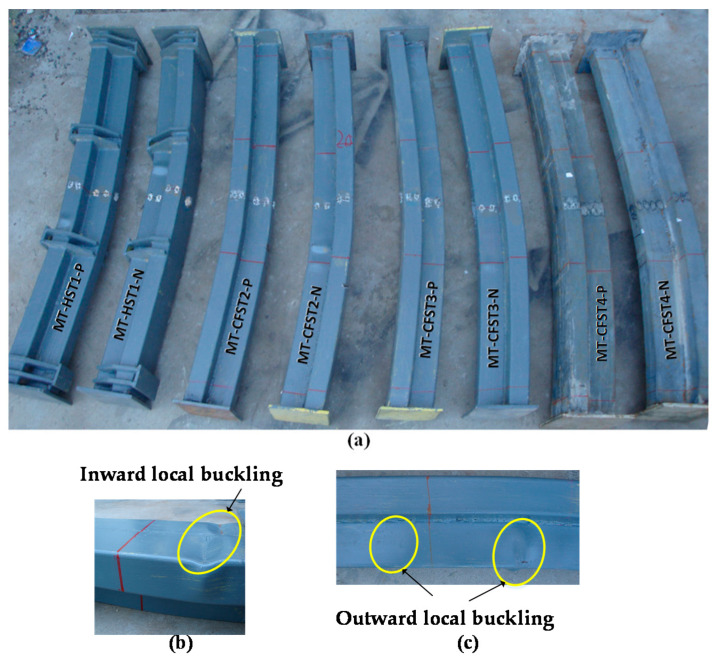
A view of specimens after testing: (**a**) failure modes of the specimens; (**b**) inward local buckling in MT-HST specimen; (**c**) outward local buckling in MT-CFST specimen.

The failure modes of all of the specimens are shown in Figure 8a. Neither brittle failure nor lateral-torsional buckling occurred throughout the loading history. MT-HST specimens suffered from severe inward local buckling (shown in Figure 8b) near the loaded points in the compression region, while some MT-CFST specimens exhibited slightly outward local buckling, as shown in Figure 8c. The difference in local buckling mode is attributed to the presence of in-filled concrete which prevents the steel tube from deforming inwards.

**Figure 9:**
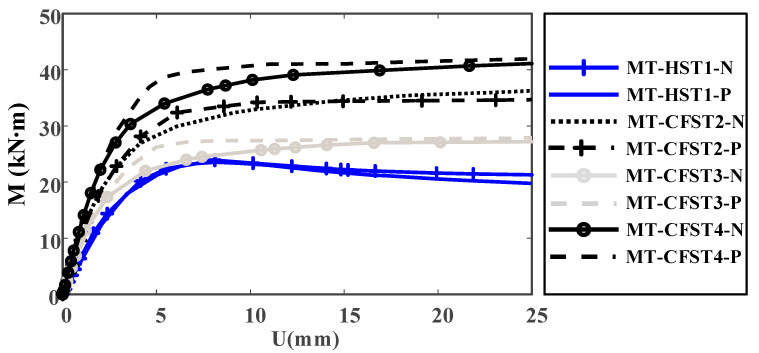
Moment-deflection curves for the tested specimens.

A plot of moment versus deflection curves for all of the tested specimens is shown in Figure 9. It was observed that MT-HST beams can develop their plastic moment resistance (22.4 kN∙m) but had limited rotation capacity because of local buckling (the MT-HST beams were not capable of maintaining plastic moment resistance when undergoing large deformation). Nearly similar moment-deflection curves were obtained for the two MT-HST beams. This is aligned with the plastic stress distribution method, in which plastic moment resistance is identical for the studied MT-HST specimen under both positive and negative bending moments.

It was observed that all MT-CFST beams exhibited very noticeable ductile behavior. A similar flexural behavior was observed for MT-CFST beams in the same group at the initial loading stage. At higher load levels, the flexural stiffness of an MT-CFST beam under a negative bending moment dropped considerably until it reached the yield plateau, in comparison with the identical one under positive bending. This may be caused by the brittle failure of concrete in the tension zone. With the initial neutral axis shifting toward the plastic neutral axis, the brittle failure area of tensile concrete under negative bending may be larger than that under positive bending. MT-CFST beams under positive bending could maintain plastic moment resistance when undergoing large deformation, while a moderate increase in resistant moment was found for the counterparts under negative bending. This is attributed to the redistribution of stress in steel and concrete and strain-hardening of steel (after yielding of steel).

Considering both the ultimate and serviceability limit states for practice, the bending moment corresponding to the strain of extreme fiber of 0.01 was defined as the ultimate bending moment (M_u-exp_), and the same approach has been adopted by Zhong [16], Han [17], Han et al. [24] and Wang et al. [26]. The values of M_u-exp_ for all of the MT-CFST specimens are shown in Table 2. It was found that M_u-exp_ for MT-CFST beams under positive bending were greater than the counterparts under negative bending. This may be explained by the Unified Theory, in which steel and concrete infill are considered as a new composite material whose compressive strength is higher than its tensile strength. For a given T-shaped section with this composite material, the theoretical flexural resistance under positive bending was greater than that under negative bending. The difference of M_u-exp_ between MT-CFST beams under positive bending and the counterparts under negative bending was within 4%.

Compared to the MT-HST specimens, the ultimate bending moment of the MT-CFST counterparts increased by 12% under positive bending and 9% under negative bending. The initial flexural stiffness of the MT-CFST beams was significantly enhanced. The results indicated that the contribution of in-filled concrete in the compression zone was significant for the flexural resistance and initial flexural stiffness of the studied composite beams since the hollow steel tubes were designed to be not susceptible to local buckling effects before developing full plastic capacity. With a reduction in the steel wall thickness, M_u-exp_ for MT-CFST3-P and MT-CFST3-N were 6.1–7.4% lower than the counterparts in the second group. It should be noted that the flexural strength and initial flexural stiffness of the composite beam may be largely controlled by the steel tube since materials lying on the outside of the composite cross section have a significant influence on the flexural behavior of the beam. The ultimate bending moments of MT-CFST specimens in the fourth group were approximately 5.7–7.9% higher than those in the second group. This demonstrated that cross-section sizes had an appreciable influence on the flexural capacity of the studied multi-cell composite T-shaped concrete-filled steel tubular specimens.

### 3.4. Neutral Axis and Strain Distributions

**Figure 10:**
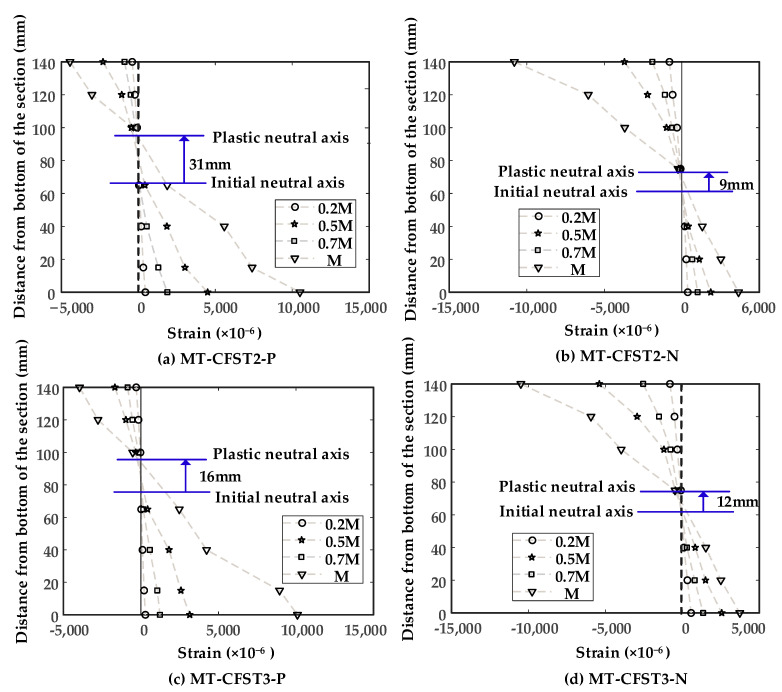
Measured strain distributions for MT-CFST specimens: (**a**) MT-CFST2-P; (**b**) MT-CFST2-N; (**c**) MT-CFST3-P; (**d**) MT-CFST3-N.

The theoretical shifting values between the initial neutral axis and plastic neutral axis are shown in Table 2. In this table, Y_PNA_ is the distance from the top of the composite section to the plastic neutral axis (PNA) corresponding to full plastic stress distribution, and Y_INA_ is the distance from the top of the composite section to the initial neutral axis (INA), which is also the unsymmetrical axis that passes through the centroid. Note that the plastic neutral axis for the MT-HST cross-section is the axis that divides the area into two equal parts, but it does not coincide with the centroidal axis. *y_s_* is the distance between PNA and INA. An upward pointing arrow (↑) means that the initial neutral axis is shifted upwards during the loading history, while a downward pointing arrow (↓) denotes downward shifting of the initial neutral axis. The measured strain distributions at four different load levels (0.2 M_u-exp_, 0.5 M_u-exp_, 0.7 M_u-exp_ and M_u_) for MT-CFST2-P and MT-CFST3-N are shown in Figure 10, where the horizontal and vertical axes represent longitudinal strain and the distance from the bottom of the composite section, respectively.

It was seen that when the bending moments were no more than 0.7 M_u-exp_, the measured longitudinal strains varied almost linearly with the distance from the initial neutral axis, which followed the plane section assumption. This indicated that the composite section works well as a unified body. Appreciable discrepancies at M_u-exp_ were observed. This was because cracking or crushing of in-filled concrete already occurred and steel tubes might experience local buckling, which resulted in stress redistribution of the composite section. From Figure 10, the initial neutral axis shifted upwards for both positive and negative bending cases, which complied with the moving tendency of the initial neutral axis predicted by the plastic stress distribution method (PSDM). The initial neutral axis moved approximately 31 mm for MT-CFST2-P and 9 mm for MT-CFST3-N, while it moved around 16 mm for MT-CFST3-P and 12 mm for MT-CFST3-N. The measured shifting values of the initial neutral axis did not agree well with the results determined by PSDM (shown in Table 2). One possible explanation is that the actual stress distribution of the composite cross-section was not identical to the assumption of the plastic stress distribution theory when the bending moment reached M_u-exp_. In addition, the inevitable error of marking the location of strain gauges also had a negative influence on the measured shifting values.

## 4. Comparison of Experimental Results against Predicted Results

**Figure 11:**
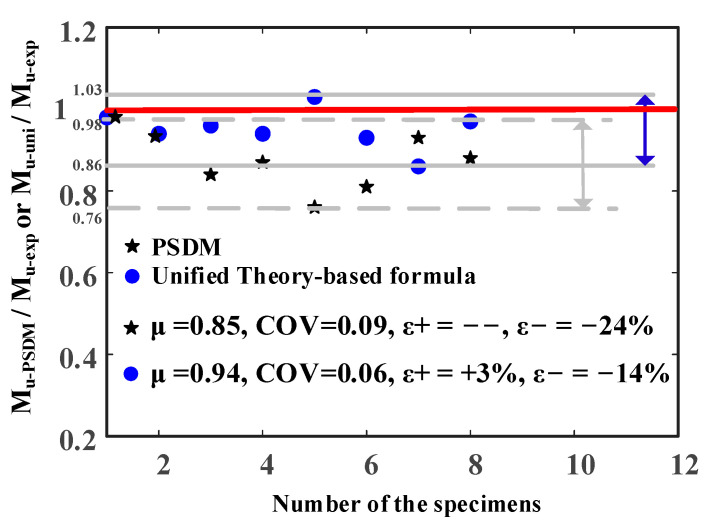
Comparison of predicted results against experimental results.

Predicted results from the plastic stress distribution method (PSDM) and the proposed formulation in line with Unified Theory are shown in Table 3. Comparison of the predicted results against experimental results is shown in Figure 11, in which the number (1, 2, 3, etc.) in the horizontal axis corresponds to the specimens shown in Table 3, for example, 1 representing MT-HST1-P, 2 representing MT-HST1-P, and so on. In Figure 11, M_u-exp_, M_u-PSDM_ and M_u-uni_ denote bending resistance obtained from the test, PSDM and the Unified Theory-based formula, respectively; a value of M_u-PSDM_/M_u-exp_ or M_u-uni_/M_u-exp_ larger than 1 means the predicted ultimate bending moments by PSDM or the Unified Theory-based formula are overestimated compared against experimental results. μ and COVs are mean value and coefficients of variation, respectively. ε+ and ε− are the maximum error of overestimation and the maximum error of underestimation, respectively. Note that for the two MT-HST beams, M_u-PSDM_, determined by the plastic section modulus times the yield strength of steel, was equal to M_u-uni_.

It was seen that both M_u-PSDM_ and M_u-uni_ for MT-HST (hollow steel tubular) beams were lower than the experimental results (M_u-exp_). This may be attributed to the strain-hardening effects of steel. For the MT-CFST beams, mean values (μ) of M_u-PSDM_/M_u-exp_ and M_u-uni_/M_u-exp_ were 0.85 and 0.94, respectively, while the coefficients of variation (COV) of M_u-PSDM_/M_u-exp_ and M_u-uni_/M_u-exp_ were 0.09 and 0.06, respectively. It demonstrated that both PSDM and the Unified Theory-based formula gave conservative predictions. Nevertheless, high values of conservative error were observed in the predicted results from PSDM, in which the conservative error was higher than 15% for some MT-CFST specimens, for example, up to a maximum of 24% for MT-CFST3-P. The conservative error for PSDM may be resulted from neglecting the benefit of stress redistribution in steel and concrete and strain-hardening effects of steel.

Compared to PSDM, the Unified Theory-based formula generally produced more reasonable conservative predictions (errors of no more than 14%) for the studied beams except for MT-CFST3-N. Although the proposed formula overestimated the bending resistance of MT-CFST3-N by 3%, the unconservative error was low to a reasonable level. This demonstrated that the Unified Theory-based formula provided improved estimation for the studied MT-CFST beams. Since the accuracy of the Unified Theory-based formula largely depends on the calculation of compressive strength (*f_sc_*) of an MT-CFST section, the results also indicated that the proposed formulation (Equation (20)) for *f_sc_*, in which shape effects on confinement to in-filled concrete were accounted for by means of introducing the modified confinement effect factor, was reasonably accurate for the MT-CFST sections.

## 5. Verification by Numerical Results

In conjunction with the experimental study, verification of the Unified Theory-based formula and PSDM for predicting the flexural strength of MT-CFST beams was conducted through finite element (FE) modelling. Numerical results from FE modelling were used to evaluate the accuracy of the proposed formulations.

### 5.1. Development and Validation of the Finite Element Model

**Figure 12:**
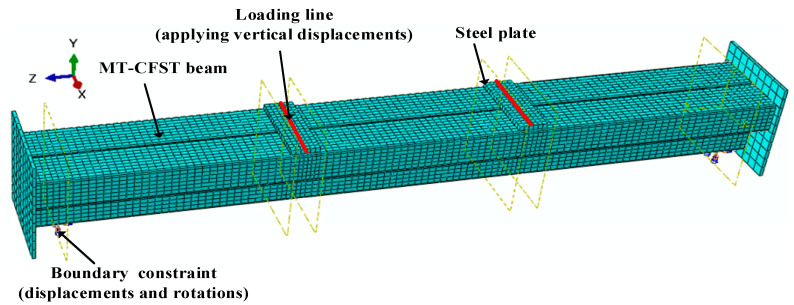
A typical FE model for MT-CFST beam.

The structural behavior of MT-CFST beams subjected to pure bending was simulated using the finite element (FE) software Abaqus 6.13 [33]. A four-node shell element with reduced integration (S4R) was used to model the steel tube, while the in-filled concrete was modeled using an eight-node brick element with reduced integration (C3D8R). The interaction between steel tube and in-filled concrete was defined through a surface-to-surface contact that used the “Hard contact” function in the normal direction and the “Coulomb friction” function in the tangential direction. Since no weld failure was observed in the test, “Tie constraint” was employed to model the interaction of steel tubes. Vertical displacements were applied at the lines corresponding to the quartering points of the beam. The displacements and relevant rotations were restrained at the two boundary lines of the beam. An appropriate mesh size was selected based on mesh convergence studies. To be consistent with the test specimens, corresponding stiffeners were created for the FE models of the two T-shaped hollow steel tubular (MT-HST) beams, while the FE models for T-shaped concrete-filled steel tubular (MT-CFST) beams did not have stiffeners. A typical FE model for the MT-CFST beam is shown in Figure 12.

The stress–strain relationship of steel tube was modelled based on the five-stage (elastic, elastic–plastic, plastic, hardening and fracture) curve suggested by Han et al. [34]. The Von Mises yield criterion in conjunction with plastic flow rules were used in multi-axial stress states. Young’s modulus and the Poisson’s ratio were taken as 2 × 10^5^ MPa and 0.3, respectively. The adopted stress–strain curve for steel tubes is given by

$$\sigma_{s}=\left\{\begin{array}{c} \;E\varepsilon_{s}\;\varepsilon_{s}\leq \varepsilon_{p}\;\left(24\right)\;\\ -A\varepsilon_{s}{}^{2}+B\varepsilon_{s}+C\;\varepsilon_{p}<\varepsilon_{s}\leq \varepsilon_{y}\;\left(25\right)\;\\ \;f_{y}\;\;\varepsilon_{y}<\varepsilon_{s}\leq \varepsilon_{uy}\;\left(26\right)\;\\ \;f_{y}+\frac{f_{u}-f_{y}}{\varepsilon_{u}-\varepsilon_{uy}}\left(\varepsilon_{s}-\varepsilon_{uy}\right)\;\varepsilon_{uy}<\varepsilon_{s}\leq \varepsilon_{u}\;\left(27\right)\;\\ f_{u}\;\varepsilon_{u}<\varepsilon_{s}\;\left(28\right) \end{array}\right.$$

where *σ_s_* and *ε_s_* are the stress and strain of steel, respectively; *E*, *f_p_*_,_ *f_y_* and *f_u_* are Young’s modulus, proportional limit, yield strength and ultimate strength of the steel, respectively; *ε_p_* = 0.8*f_y_*/*E*; *ε_y_* = 1.5*ε_p_*; *ε_uy_* = 10*ε_y_*; *ε_u_* = 100*ε_y_*; *A* = 0.2*f_y_*/(*ε_y_* − *ε_p_*)^2^; *B* = 2*Aε_y_*; *C* = 0.8*f_y_* + *Aε_p_*^2^ − *BAε_p_*.

The concrete damaged plasticity model [35] combined with the stress–strain relationship suggested by Han et al. [34] were used to represent the mechanical behavior of concrete. The adopted stress–strain curve for infilled concrete is given by

$$y=\left\{\begin{array}{c} 2x-x^{2}\;\left(x\leq 1\right)\;\left(29\right)\;\\ \frac{x}{\beta {\left(x-1\right)}^{\eta }+x}\;(x>1)\;\left(30\right)\; \end{array}\right.$$

where *x* = *ε_c_* /*ε*_0_; *y* = *σ_c_* /*σ*_0_; *σ*_0_ = *f_c_**^’^***; *ε*_0_ = *ε_c_*_0_ + 800*ξ*^0.2^ × 10^−6^; *ε_c_*_0_ = (1300 + 12.5*f_c_**^’^***) × 10^−6^; *η* = 1.6 + 1.5/*x*; *β* = (*f_c_**^’^***)^0.1^/[1.2 × (1 + *ξ*)^0.5^]; *σ_c_* and *ε_c_* are the stress and strain of the concrete, respectively; *f_c_**^’^*** is the compressive strength of the concrete, and *ξ* is the confinement effect factor.

To appropriately consider the confinement effect of the multi-cell T-shaped section, the confinement effect factor (*ξ*) in the above equations was replaced by the modified confinement effect factor (*ξ_MT_*) proposed in the present paper. For the validation of created FE models, stress–strain relationships obtained from material tests were used.

**Figure 13:**
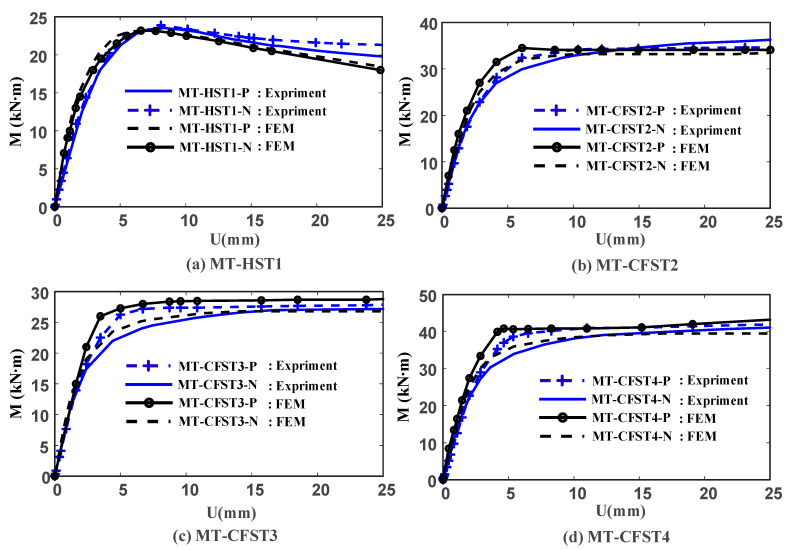
Comparison of numerical results with test results: (**a**) MT-HST1; (**b**) MT-CFST2; (**c**) MT-CFST3; (**d**) MT-CFST4.

A comparison of numerical results from the developed FE models against test results was shown in Figure 13. It is seen that the test results were generally replicated accurately, though slight discrepancies in flexural stiffness (strength) can be seen between the numerical results and test results.

### 5.2. Comparison of Predicted Results and Numerical Results

A series of FE models with a wider range of cross-section sizes, wall thicknesses (*t*), yield stresses (*f_y_*) and compressive strengths (*f_c_*^’^) were studied. The height-to-width ratio (*a* + *b*/2*c*) of the composite T-shaped section varied from 0.8 to 2.5, and t ranged from 3 to 7mm. Yield stress (*f_y_*) of 345–500 MPa and compressive strength (*f_c_^’^*) of 30–85 MPa were considered. Both positive and negative bending moments were considered for two identical FE models. A total of 52 FE models were created. A summary of the FE models used for verification is shown in Table 4. The primary variables in the first group were height-to-width ratio, wall thickness, yield strength and compressive strength. The main differences among numerical models in the second group were wall thickness and yield strength, while the main variables were height-to-width ratio, wall thickness and compressive strength for the third group. Details on the geometric and material properties of the FE models are provided in Table A1 of Appendix B.

**Figure 14:**
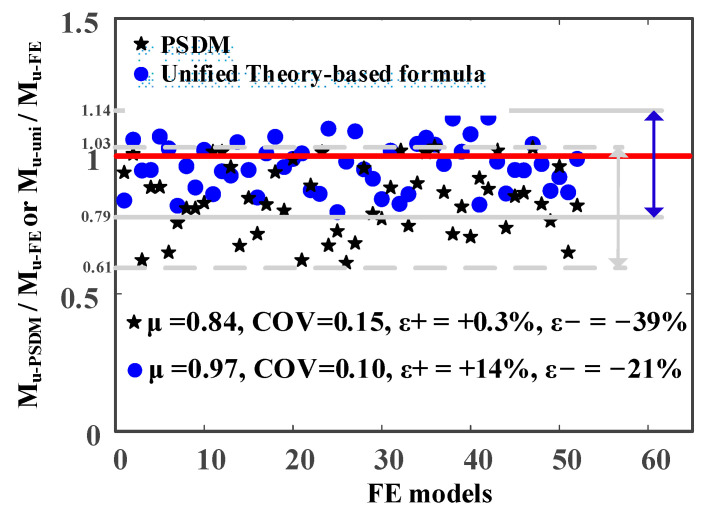
Comparison of predicted results (from PSDM and Unified Theory) against numerical results.

A comparison of the predicted results against the numerical results is shown in Figure 14, where the number in the horizontal axis corresponds to the specific FE model (for example, 10 representing the tenth FE model). In Figure 14, μ and COV denote mean value and coefficients of variation, respectively; M_u-FE_, M_u-PSDM_ and M_u-uni_ are ultimate bending moments determined by FE modelling analysis, PSDM and the Unified Theory-based formula, respectively; and ε+ and ε− are the maximum error of overestimation and the maximum error of underestimation, respectively. A value of M_u-PSDM_/M_u-FE_ or M_u-uni_/M_u-FE_ larger than 1 means that the ultimate bending moments determined by PSDM or the Unified Theory-based formula are overestimated compared against numerical results.

From Figure 14, the mean values of M_u-PSDM_/M_u-FE_ and M_u-uni_/M_u-exp_ were 0.81 and 0.97, respectively, while the COVs of M_u-PSDM_/M_u-FE_ and M_u-uni_/M_u-exp_ were 0.10 and 0.16, respectively. This shows that the results determined by the Unified Theory-based formula had lower deviation from numerical results compared to PSDM. It was seen that PSDM gave safe predictions but produced large conservative errors for some cases, while the Unified Theory-based formula provided accurate and safe results for most FE models. The Unified Theory-based formula produced results with unconservative errors (within 14%) for a few FE models. From the perspective of practice, the unconservative errors were still acceptable since safety factors of no less than 1.10 are commonly used in design. The maximum error of underestimation of M_u-FE_ was 39% for PSDM and 21% for the Unified Theory-based formula. The results indicate that the Unified Theory-based formula provided improved estimations in comparison to PSDM.

## 6. Conclusions

The flexural strength of MT-CFST beams was investigated in this paper. A Unified Theory-based formula applicable to MT-CFST beams was developed by means of introducing a modified confinement effect factor that accounts for the geometry effects on confinement to in-filled concrete. A series of experimental and numerical studies were carried out. The accuracy of the Unified Theory-based formula and the plastic stress distribution method (PSDM) was verified against the test results and numerical results. Based on the experimental observations and numerical analysis, the following conclusions were made:

(1) All the MT-CFST beams exhibited very noticeable ductile behavior. The flexural strength of MT-CFST specimens under positive bending was higher than the counterparts under negative bending. This was aligned with the Unified Theory in which the T-shaped cross section was taken as one new composite material whose compressive strength was greater than its tensile strength. Increasing in-filled concrete and cross-section sizes had an appreciable influence on the flexural strength of the studied MT-CFST specimens.
(2) Both the Unified Theory-based formula and PSDM generally gave conservative predictions. Predicted results from the Unified Theory-based formula were in good agreement with both experimental results and numerical results. The Unified Theory-based formula produced reasonably conservative predictions for most MT-CFST beams and gave acceptable unconservative errors for few MT-CFST beams. PSDM provided safe predictions but produced large conservative errors in some cases.

## Figures and Tables

**Table 1:** Geometric and material properties of the specimens.

Specimen	Group	*a* (mm)	*b* (mm)	*c* (mm)	*t* (mm)	R-_in_ (mm)	L (mm)	E_s_ (GPa)	*f_y_* (MPa)	E_c_ (GPa)	*f_c_*^′^ (MPa)
MT-HST1-P	1	60.1	80.3	79.9	2.48	3.97	1300.5	198.2	315		
MT-HST1-N		60.4	79.8	80	2.50	4.75	1301	198.2	315		
MT-CFST2-P	2	59.9	80	80.4	2.52	5.25	1300	198.2	315	35.5	41.3
MT-CFST2-N		60.5	80.2	79.6	2.46	5.09	1299.6	198.2	315	35.5	41.3
MT-CFST3-P	3	59.8	79.2	79.9	1.99	3.38	1299.8	199.6	321.6	35.5	41.3
MT-CFST3-N		59.5	80.3	79.6	2.01	4.42	1299.4	199.6	321.6	35.5	41.3
MT-CFST4-P	4	60.3	100.7	80.4	2.52	5.80	1300.9	198.2	315	35.5	41.3
MT-CFST4-N		59.8	100.1	79.7	2.49	4.01	1301.2	198.2	315	35.5	41.3

**Table 2:** Ultimate bending moment and shifting values between initial neutral axis and plastic neutral axis.

Specimen	Group	M_u-exp_ (kN∙m)	Y_INA_ (mm)	Y_PNA_ (mm)	*y_s_* (mm)
MT-HST1-P	1	22.8	53.50	57.89	4.39↓
MT-HST1-N		23.9	86.46	82.11	4.35↑
MT-CFST2-P	2	34.7	53.19	33.50	19.69↑
MT-CFST2-N		33.0	86.18	80.56	5.62↑
MT-CFST3-P	3	27.3	52.93	29.71	23.22↑
MT-CFST3-N		26.9	86.81	79.95	6.83↑
MT-CFST4-P	4	42.6	61.15	35.86	25.29↑
MT-CFST4-N		38.7	99.26	101.44	2.18↓

**Table 3:** Predicted results from PSDM and the Unified Theory-based formula.

Specimen	Group	M_u-exp_ (kN∙m)	M_u-PSDM_ (kN∙m)	M_u-uni_ (kN∙m)	M_u-PSDM_/M_u-exp_	M_u-uni_/M_u-exp_
MT-HST1-P	1	22.8	22.4	22.4	0.98	0.98
MT-HST1-N		23.9	22.4	22.4	0.94	0.94
MT-CFST2-P	2	34.7	29	33.2	0.84	0.96
MT-CFST2-N		33	28.6	31.1	0.87	0.94
MT-CFST3-P	3	27.3	20.8	27.8	0.76	1.03
MT-CFST3-N		26.9	21.9	25	0.81	0.93
MT-CFST4-P	4	42.6	39.8	35.8	0.93	0.86
MT-CFST4-N		38.7	34.1	37.6	0.88	0.97
μ					0.85	0.94
COV					0.09	0.06
ε+					--	+3%
ε−					−24%	−14%

**Table 4:** A summary of the FE models used for verification.

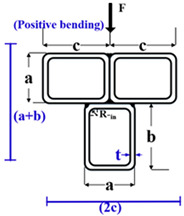 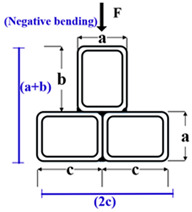	**Group**	**(*a* + *b*)/2*c***	***t* (mm)**	***f_y_* (MPa)**	***f_c_^’^* (MPa)**	**Moment**	**Number of Models**
	1	0.8 2.0	3 4	345	30 65	Positive and negative	24
	2	1.5	4 6	300 425 500	45	Positive and negative	12
	3	1.2 2.5	5 7	450	60 85	Positive and negative	16

## Appendix A

The equilibrium equations used to obtain *y_p_* are given by

(A1) $$f_{y}A_{s}=f_{y}A_{s}^{\prime }+0.85f_{c}A_{c}^{\prime }$$

where

(A2) $$A_{s}=4t\left(y_{p}-b-t\right)+2ct+[ab-\left(a-2t\right)\left(b-2t\right)$$

(A3) $$A_{s}^{\prime }=4t\left(a+b-y_{p}-t\right)+2ct$$

(A4) $$A_{c}^{\prime }=\left(a+b-y_{p}-t\right)\left(2c-4t\right)$$

*y_c_* (in Figure 4) is given by

(A5) $$y_{c}=\frac{a+b+y_{p}-t}{2}$$

*y_s_* (in Figure 4) is given by

(A6) $$y_{s}=y_{1}-y_{2}$$

where

(A7) $$y_{1}=\frac{\frac{4t\left(y_{p}-b-t\right)\left(b+t+y_{p}\right)}{2}+2ct\left(b+\frac{t}{2}\right)+\frac{\left[ab-\left(a-2t\right)\left(b-2t\right)\right]b}{2}}{4t\left(y_{p}-b-t\right)+2ct+\left[ab-\left(a-2t\right)\left(b-2t\right)\right]\;}$$

(A8) $$y_{2}=\frac{\frac{4t\left(a+b-y_{p}-t\right)\left(a+b-y_{p}-t\right)}{2}+2ct\left(a+b-\frac{t}{2}\right)}{4t\left(a+b-y_{p}-t\right)+2ct}$$

## Appendix B

**Table A1:** Details of the geometric and material properties of the numerical models (P and N denote positive and negative bending moment, respectively).

Group	(a + b)/2c	t (mm)	*f_y_* (MPa)	*f_c_’* (MPa)	M	Group	(a + b)/2c	t (mm)	*f_y_* (MPa)	*f_c_’* (MPa)	M
1	0.8	3	235	30	P	2	1.5	4	425	45	P
	0.8	3	235	30	N		1.5	4	425	45	N
	0.8	3	235	65	P		1.5	4	500	45	P
	0.8	3	235	65	N		1.5	4	500	45	N
	0.8	3	345	30	P		1.5	6	300	45	P
	0.8	3	345	30	N		1.5	6	300	45	N
	0.8	3	345	65	P		1.5	6	425	45	P
	0.8	3	345	65	N		1.5	6	425	45	N
	0.8	4	235	30	P		1.5	6	500	45	P
	0.8	4	235	30	N		1.5	6	500	45	N
	0.8	4	235	65	P	3	1.2	5	450	60	P
	0.8	4	235	65	N		1.2	5	450	60	N
	0.8	4	345	30	P		1.2	5	450	85	P
	0.8	4	345	30	N		1.2	5	450	85	N
	0.8	4	345	65	P		1.2	7	450	60	P
	0.8	4	345	65	N		1.2	7	450	60	N
	2.0	3	235	30	P		1.2	7	450	85	P
	2.0	3	235	30	N		1.2	7	450	85	N
	2.0	3	235	65	P		2.5	5	450	60	P
	2.0	3	235	65	N		2.5	5	450	60	N
	2.0	3	345	30	P		2.5	5	450	85	P
	2.0	3	345	30	N		2.5	5	450	85	N
	2.0	3	345	65	P		2.5	7	450	60	P
	2.0	3	345	65	N		2.5	7	450	60	N
2	1.5	4	300	45	P		2.5	7	450	85	P
	1.5	4	300	45	N		2.5	7	450	85	N
